# Relationship between tear film stability, dry eye symptoms, and night driving vision among Malaysian adults

**DOI:** 10.1371/journal.pone.0320223

**Published:** 2025-03-26

**Authors:** Pui Juan Woi, Purani Pathmanathan, Selena Yi Han Sieh, Mohd Harimi Abd Rahman, Haliza Abdul Mutalib, Madhavendra Bhandari, Naufal Nordin, Wan Muhammad Hirzi Wan Din

**Affiliations:** 1 Optometry and Vision Science Program, Center for Community Health Studies (ReaCH), Universiti Kebangsaan Malaysia, Kuala Lumpur, Malaysia; 2 Optometry and Vision Science Program, Center for Rehabilitation & Special Needs Studies (iCaRehab), Universiti Kebangsaan Malaysia, Kuala Lumpur, Malaysia; 3 School of Optometry, Faculty of Medicine & Health Sciences, UCSI University, Cheras, Kuala Lumpur, Malaysia; University of Houston College of Optometry, UNITED STATES OF AMERICA

## Abstract

Background

Dry eye disease, a prevalent condition globally, affects the quality of the tear film and, subsequently, vision, especially during visually demanding tasks like driving at night. This study aims to evaluate the relationship between tear film stability, dry eye symptoms, and self-reported difficulties in night driving among Malaysian adults. Methods: Ninety participants aged 18-40 years with at least one year of night driving experience were recruited. Tear film stability was assessed using non-invasive tear break-up time (NIBUT), while dry eye symptoms were measured with the Ocular Surface Disease Index (OSDI). Night driving vision difficulties were evaluated using the Vision and Night Driving Questionnaire (VND-Q). Results: Participants with shorter NIBUT (mean =  3.95 ±  1.32 s; median =  3.97 s, IQR: 2.87-5.03 s) reported significantly greater difficulties in night driving compared to those with normal NIBUT (mean =  9.80 ±  3.86 s; median =  8.23 s, IQR: 6.90-11.70 s) (p <  0.001). Similarly, participants with severe dry eye symptoms had higher VND-Q scores (mean rank =  76.75) compared to those with asymptomatic to moderate symptoms (mean rank =  35.68–44.03) (p <  0.001). Spearman’s correlation showed moderate negative associations between NIBUT (r =  -0.327), OSDI (r =  -0.538), and VND-Q scores. However, there was no significant correlation between NIBUT and OSDI score. Multiple regression analysis revealed that both NIBUT and OSDI significantly predicted the VND-Q score, explaining 43.2% of the variance (p <  0.001). Conclusions: The findings suggest that poor tear film stability and severe dry eye symptoms contribute to night driving difficulties. Future studies should explore interventions aimed at improving tear film stability and dry eye symptoms to enhance driving safety at night.

## Introduction

Dry eye disease has become a common reason for seeking eye care, emerging as a significant modern eye health issue. This multifactorial condition arises from an imbalance in the tear film and ocular surface, leading to ocular surface dysfunction and a range of symptoms [1,2]. Despite its prevalence, estimated to affect between 5% and over 50% of adults globally [3], dry eye is often overlooked as it is not a common cause of significant visual morbidity and is not regularly assessed during routine eye examinations. The condition presents with varied symptoms, such as tearing, burning, stinging, foreign body sensation, photophobia, and blurred vision [4,5]. Recent studies show that 15–33% of adults experience symptomatic dry eye, with higher prevalence observed in older adults, females, and those of Asian descent [6]. While dry eye may not directly cause severe visual impairment, it can degrade the quality of vision due to irregularities in the tear film and refracting surfaces of the eye [7,8].

Given that dry eye significantly impacts daily activities and quality of life [5,9,10], it can also affect essential functions such as driving. Driving is an integral part of daily life for many people worldwide. While visual acuity is the standard screening test when applying for a driver’s license, numerous other aspects of visual function undoubtedly play a role in ensuring effective vehicle control [11]. A study conducted in China revealed that the frequencies of unsafe driving habits and performance are significantly higher in drivers with dry eye disease, which may increase the risk of dangerous driving [12]. Another study also reported that drivers with dry eye disease more commonly missed targets at crossroad entrances than targets appearing straight ahead as compared with drivers without dry eye disease [13].

Driving is a crucial daily activity in modern life, including night driving. Approximately one-third of adults report difficulty seeing at night while driving [14,15], with vision problems frequently cited as the primary cause [16]. The reduced light levels on roadways at night exacerbate these issues, making visibility more challenging [15,17], and these difficulties are likely to be compounded in individuals with dry eye disease. Despite the commonality of these challenges, limited research has focused on how dry eye symptoms—while not necessarily impacting visual acuity during clinical assessments—may impair vision in night driving situations.

Although the public and eye care providers often recognise dry eye symptoms, their impact on night driving vision beyond decreased visual acuity remains poorly understood. This study seeks to address this gap by examining the relationship between dry eye symptoms and night driving vision using both objective and subjective evaluations. Tear film stability was measured using non-invasive tear break-up time (NIBUT), while self-reported dry eye symptoms and night driving difficulties were assessed through validated questionnaires. Tear film stability refers to the ability of the tear layer to maintain a smooth optical surface, whereas NIBUT quantifies how long the tear film remains intact before breaking up. The NIBUT assessment provides a quantifiable indicator of tear film stability, which is crucial for visual quality, particularly during night driving. This study provides valuable insights for eye care providers and patients, enhancing the understanding of how dry eye symptoms influence night driving performance and highlighting potential implications for vision care and road safety.

## Materials and methods

Ninety participants were recruited using the purposive sampling method in this cross-sectional study, conducted from 2nd May 2022 to 1st September 2022. The sample size for this study was determined using G * Power software [18]. A small effect size (f² =  0.2) was selected to ensure the detection of clinically meaningful relationships between tear film stability, dry eye symptoms, and night driving vision. A power of 0.95 was chosen to minimize the risk of Type II errors, given the importance of accurately identifying significant predictors in the context of visual health and driving safety among Malaysian adults. A significance level (α) of 0.05 was used, and the calculation was performed for multiple linear regression analysis. The initial sample size calculation indicated a requirement of 81 participants. To account for potential dropout, an additional 10% were included, resulting in a final target sample size of 90 participants.

This study was conducted at the Optometry Clinic of the National University of Malaysia. The participants were aged between 18 and 40 years old (mean age =  24.78 ±  4.52 years), a group representing individuals actively engaged in driving in Malaysia [19]. This was selected to control for age-related visual decline that may confound results. All of them had at least one year of driving experience with an active driving licence and drove at night at least once a week. All participants underwent a comprehensive ocular health examination to ensure no ocular pathologies (except dry eye disease) and binocular anomalies were present. None had a history of ocular diseases, including binocular anomalies or amblyopia (except dry eye disease), and a history of systemic diseases or medication with known ocular involvement. All participants had their habitual refractive correction with distance visual acuity of 6/6 or better in both eyes. The study was approved by the Research Ethics Committee of the National University of Malaysia (UKM PPI/111/8/JEP-2022-103) and conformed to the tenets of the Declaration of Helsinki involving human participants. All participants gave written informed consent before taking part in the study.

Demographic data, including sex, ethnicity, average hour of night driving in a 24-hour period, vehicle type, and optical correction type, were collected for each participant. The Vision and Night Driving Questionnaire (VND-Q) assessed participants’ self-reported night driving difficulties [20]. The VND-Q contains nine reliable, unidimensional questions that enable measurement of the level of nighttime vision difficulties reported by drivers. The score of each question was calculated using a five-point Likert-type scale, with responses ranging from one =  ‘no difficulty’ to five =  ‘extreme difficulty’. The ordinal VND-Q score was converted to a Rasch-scaled interval level score ranging from zero to ten. Higher scores indicated more vision-related night driving difficulties.

The Ocular Surface Disease Index (OSDI) questionnaire was used to evaluate participants’ dry eye symptoms subjectively [21]. The OSDI questionnaire is a 12-item instrument created to evaluate subjective dry eye symptoms and their effects on the vision-related activities of daily living within the previous week. This questionnaire has three subscales: ocular symptoms (three questions), vision-related functions (six questions), and environmental triggers (three questions). The score was graded on a five-point Likert-type scale, with responses ranging from zero =  “none of the time” to four =  “all of the time”. The OSDI total score ranged from zero to 100 points and was obtained by multiplying the total score of all the questions by 25 and dividing the result by the number of valid answers. The OSDI total score was used to categorise the participants’ dry eye symptoms as asymptomatic (0–12 points), mild (13–22 points), moderate (23–32 points), or severe (33–100 points) [22].

NIBUT was measured to evaluate the tear film stability. NIBUT is the interval that elapses between a complete blink and the appearance of the first break in the tear film [23]. The same researcher performed all NIBUT measurements in this study. NIBUT was measured using Tearscope Plus (Keeler, Windsor, UK), which is designed to be used with a slit-lamp biomicroscope. The tearscope was held close to the participant’s eye and positioned to allow observation through the sight hole through one of the biomicroscope objectives. An illuminated grid pattern was projected onto the precorneal tear film, and the participants were instructed to blink a few times before holding still to take the NIBUT measurement. If the participant blinked before the point of NIBUT, the time of the blink was recorded. The average of three NIBUT measurements on each eye was taken.

The statistical analysis was performed using Statistical Package for Social Sciences (SPSS) version 28.0 (IBM Corp, Armonk, NY). The normality of data was tested with the Shapiro-Wilk test. All data sets were not normally distributed. The Wilcoxon Signed Rank test was conducted to compare the differences in NIBUT between the right and left eyes among participants. The Mann-Whitney U and Kruskal-Wallis tests were performed to evaluate the differences between NIBUT and demographic variables. The differences in VND-Q scores between low and normal NIBUT groups were analysed by the Mann-Whitney U test, while the differences in VND-Q scores between different severities of the OSDI categories were analysed by the Kruskal-Wallis test. The prediction of the VND-Q score from the NIBUT and OSDI score was tested using multiple regression. The assumptions of multiple regression (independence and collinearity) were checked to ensure a valid interpretation of the results. All p-values were 2-sided, and a statistical level of p <  0.05 was considered significant.

## Results

In this study, NIBUT of the right eye was used for data analysis as there was no significant difference between NIBUT of the right (mean =  6.88 ±  4.11 s; median =  6.02 s, IQR =  3.98-8.21 s) and left eyes (mean =  7.67 ±  4.56 s; median =  6.60 s, IQR =  4.21-9.65 s), (T =  2504.0, z =  -1.837, N =  90, p =  0.066). The demographic categorical data are presented in Table 1. No statistically significant difference in NIBUT was found between sex, ethnicity, vehicle type, and optical correction type for night driving.

**Table 1:** Demographic categorical data of the study population.

Categorical variable		Total (N = 90)	p-value
**Sex**	Male	40 (44%)	0.090
	Female	50 (56%)	
**Ethnicity**	Malay	47 (52%)	0.060
	Indian	29 (32%)	
	Chinese	14 (16%)	
**Vehicle type**	Car	75 (83%)	0.878
	Motorcycle	14 (15%)	
	Both	1 (2%)	
**Optical correction type for night driving**	Glasses	55 (61%)	0.885
	Contact lenses	3 (3%)	
	Not required	32 (36%)	

The median NIBUT value of the right eye, 6.02 s, was used as the cut-off value to categorise the participants into short and normal NIBUT groups. Each group’s NIBUT and VND-Q score are listed in Table 2. The NIBUT of the short NIBUT group (mean rank =  23.0, n =  45) was significantly shorter than the normal NIBUT group (mean rank =  68.0, n =  45), U =  2025.0, z =  8.171, p <  0.001. The VND-Q score of the short NIBUT group (mean rank =  52.87, n =  45) was significantly higher than the normal NIBUT group (mean rank =  38.13, n =  45), U =  681.0, z =  -2.683, p =  0.007. The result indicates that individuals with short NIBUT tend to report greater difficulties in night driving.

**Table 2:** Comparison of NIBUT and VND-Q score between participants with short and normal NIBUT, with a cut-off of ≥  6.02 s for normal NIBUT.

	NIBUT category				p-value
	Short, < 6.02 s (n = 45)		Normal, ≥ 6.02 s (n = 45)		
	Mean ± SD	Median (IQR)	Mean ± SD	Median (IQR)	
**NIBUT (s)**	3.95 ± 1.32	3.97 (2.87-5.03)	9.80 ± 3.86	8.23 (6.90-11.70)	< 0.001 *
**VND-Q score**	3.13 ± 1.45	3.03 (2.21-4.09)	2.35 ± 1.55	2.21 (1.46-3.26)	0.007 *

*statistical significance.

The OSDI and VND-Q scores for each severity of the OSDI category are listed in Table 3. The OSDI scores significantly differed across all categories, X^2^(3) =  79.37, p <  0.001. There was a statistically significant difference in VND-Q scores between different severities of the OSDI category, X^2^(3) =  25.651, p <  0.001, with the mean rank score of 35.68 for the asymptomatic group, 44.03 for the mild group, 42.17 for the moderate group, and 76.75 for the severe group. The post-hoc analysis indicated that the severe OSDI group had a significantly higher VND-Q score than the other three OSDI groups. These results suggest that individuals with severe dry eye symptoms tend to report greater difficulties in night driving.

**Table 3:** Comparison of OSDI and VND-Q scores between different severities of OSDI category.

Score	OSDI category								p-value
	Asymptomatic (n = 37)		Mild (n = 30)		Moderate (n = 9)		Severe (n = 14)		
	Mean ± SD	Median (IQR)	Mean ± SD	Median (IQR)	Mean ± SD	Median (IQR)	Mean ± SD	Median (IQR)	
OSDI	5.86 ± 3.31	6.30 (2.10-8.30)	16.04 ± 2.64	16.70 (14.58-18.80)	27.57 ± 2.93	27.10 (25.0-29.20)	43.01 ± 8.56	41.70 (35.93-45.30)	< 0.001 *
VND-Q	2.15 ± 1.38	2.21 (1.46-3.03)	2.61 ± 1.24	2.77 (1.96-3.48)	2.50 ± 1.31	2.77 (1.46-3.03)	4.71 ± 1.16	4.79 (3.95-5.57)	< 0.001 *

*statistical significance.

The Spearman’s rank-order correlation was used to examine the correlation between the participants’ VND-Q, NIBUT and OSDI scores, as shown in Table 4. The results showed that NIBUT (r =  -0.327) and OSDI score (r =  -0.538) significantly correlated with VND-Q score. Both correlations were moderately strong and negatively correlated. The correlation between NIBUT and OSDI score was also investigated, but it was found to be weak and insignificant (r =  -0.196).

**Table 4:** Correlation between VND-Q, NIBUT, and OSDI score.

Bivariate correlation	Spearman’s rank-order correlation, r	p-value
**VND-Q score and NIBUT**	-0.327	0.002 *
**VND-Q and OSDI scores**	-0.538	<0.001 *
**NIBUT and OSDI score**	-0.196	0.064

*statistical significance.

A multiple linear regression was run to predict the VND-Q score from the NIBUT and OSDI score. The results of the regression showed that the model explained 43.2% of the variance (adjusted =  41.9%), and the model was a significant predictor of VND-Q score, F(2, 87) =  33.056, p <  0.001. The NIBUT (B =  -0.096, p =  0.003) and OSDI score (B =  0.061, p <  0.001) contributed significantly to the prediction. The final predictive model was VND-Q =  2.359 – (0.096 x NIBUT) +  (0.061 x OSDI score).

## Discussion

This study provides an overview of the relationship between tear film stability, dry eye symptoms and night driving vision in Malaysian adults. The NIBUT and OSDI score were significantly related to the drivers’ self-reported vision-related night driving difficulties. These findings underscore the importance of considering dry eye factors in assessing an individual’s ability to drive safely, particularly at night.

The tear film stability of the participants was determined based on NIBUT in this study. No significant differences in NIBUT were found between the right and left eyes, nor across various demographic categories, including sex, ethnicity, vehicle type, and optical correction type for night driving. This consistency suggests that NIBUT was a stable measure in this study that was not influenced by these demographic factors. The median NIBUT value of the right eye, 6.02 s, was used as the cut-off value to divide the participants into short and normal NIBUT groups. This cut-off was chosen as it reflects the natural distribution of NIBUT values in the study population, ensuring an appropriate division for statistical analysis. Most previous studies conducted on Caucasian participants considered the NIBUT cut-off value of ≥  10 s, which is recommended by the Tear Film and Ocular Surface Society (TFOS) [24–27]. However, many Asian-based studies have shown that the NIBUT value of the Asian population is much shorter [28–31]. Given that this study sample consists of Malaysian adults, the median NIBUT of 6.02 s aligns with findings from other studies on Asian populations and provides a more relevant clinical reference. In a comparison study, Asians were found to have a lower level of tear film stability and tear osmolarity than Caucasians [32], supported by the evidence of higher meibomian gland dropout levels in Asians than Caucasians [3,32,33]. These findings justify the short NIBUT in Asians.

With a cut-off value of ≥  6.02 s for normal NIBUT, the short NIBUT group reported significantly greater night driving difficulties compared to those with normal NIBUT. The key finding of this study was that the NIBUT and OSDI scores were significantly correlated with drivers’ self-reported night driving difficulties, with both factors playing a crucial role in contributing to these challenges, as reflected in the VND-Q score. These findings are in line with previous questionnaire-based research [5,34–37] and provide added value compared to those studies since NIBUT was measured in the present study. Moreover, previous studies typically assessed night driving vision using only one or a few questions within broader quality-of-life questionnaires. In contrast, the present study utilised a validated questionnaire specifically designed to evaluate night-driving difficulties comprehensively [18].

The impacts of tear film instability on the quality of vision are widely acknowledged. Optical metrics of tear film quality and retinal image quality have been linked to the deterioration in vision associated with tear film break-up [38,39]. Tear film instability introduces surface irregularities due to a loss of homeostasis, compromising the optical pathway and leading to a decline in retinal image clarity [40,41]. A recent systematic review provides clear evidence of an association between dry eyes and higher-order aberrations (HOAs), highlighting the tear film as a crucial factor in this relationship [42]. HOAs are unlikely to cause significant visual loss; however, they can lead to image defocus and distortion, which cannot be corrected with optical aids [43]. The dim illumination during night driving may exacerbate the effects of tear film instability caused by dry eyes. At night, the pupils dilate, increasing the individual’s susceptibility to perceiving halos and other night vision disturbances when exposed to bright light sources, such as oncoming headlights [44]. Drivers’ vision is already compromised during night driving due to pupil dilation, and tear film instability may further worsen visual impairment, thereby intensifying the night driving challenges. The post-hoc analysis further confirmed that drivers with severe dry eye symptoms reported significantly greater night driving difficulties compared to those with normal to moderate dry eye symptoms, highlighting the potential impact of severe dry eye on visual performance during night driving.

Interestingly, the correlation between NIBUT and OSDI was found to be weak and insignificant. This suggests that these two measures—an objective assessment of tear film stability (NIBUT) and a subjective evaluation of dry eye symptoms (OSDI)—may capture distinct aspects of dry eye disease. A recent study with a large sample size has also reported a weak correlation between dry eye symptoms and clinical signs [45]. NIBUT primarily reflects the physical stability of the tear film, while OSDI focuses on the individual’s perception of symptoms related to dry eye, such as discomfort and visual disturbances. The lack of a significant correlation between these two measures indicates that a comprehensive diagnosis of dry eye disease may require the consideration of both objective and subjective assessments. This is particularly relevant given that no single gold standard sign or symptom has been established that perfectly correlates with dry eye disease [27]. Further supporting this notion, previous studies have demonstrated that the signs and symptoms of dry eye disease are not uniformly correlated across the broad population and often do not occur in sync [46–48]. This variability emphasises the complexity of dry eye disease. It highlights the necessity of employing a multifaceted approach for diagnosis and management, as relying solely on one type of measurement may overlook critical aspects of the condition.

The findings from this study suggest several key clinical implications for managing patients with dry eye disease, particularly those who drive at night. Eye care providers should consider incorporating both objective measures like NIBUT and subjective symptom assessments (e.g., OSDI) into routine exams for patients reporting night driving difficulties, as poor tear film stability and severe dry eye symptoms significantly impact night driving performance. Given the significant association between tear film instability and night driving difficulties, targeted interventions to improve tear film quality may enhance both visual comfort and driving safety in low-light conditions. Effective therapeutic strategies include tear substitutes, such as preservative-free artificial tears, which help stabilize the tear film and reduce fluctuations in vision during prolonged driving sessions. For individuals with aqueous-deficient dry eye, punctal plugs can be beneficial in retaining natural tears and prolonging tear film stability. Environmental modifications, such as using in-car humidifiers to reduce tear evaporation, limiting prolonged screen time before driving, and adopting proper blinking techniques, can further support tear film integrity. Additionally, educating patients about the link between dry eye and driving safety is essential, along with encouraging precautions, such as using lubricating drops before driving or avoiding night driving in severe cases.

Despite these insights, several limitations of the study should be considered. Notably, the VND-Q is a self-reported measure which can be influenced by individual perceptions and biases. Individuals may perceive their night driving difficulties differently due to personal experiences or external factors, potentially influencing their responses. This introduces inherent subjectivity and the possibility of response bias. To address this limitation, future studies should incorporate objective assessments, such as night driving simulators, real-world night driving evaluations, or eye-tracking technology, to provide a more comprehensive understanding of how tear film instability impacts night driving performance. These objective methods would complement self-reported data and reduce bias in assessing the real-world implications of dry eye on night driving. This study focused on Malaysian adults aged 18–40 years, which limits its generalizability to older drivers. Older individuals are more likely to experience age-related changes in tear film stability, meibomian gland dysfunction, and higher-order visual disturbances, all of which may exacerbate night-driving difficulties [49,50]. Given that older drivers are at higher risk for dry eye disease, future research should include a broader age range to determine whether similar associations between tear film stability and night driving difficulties persist across different age groups. Additionally, the cross-sectional nature of this study limits the ability to draw causal inferences. Longitudinal studies would be beneficial to examine whether improvements in tear film stability and dry eye symptoms lead to corresponding reductions in night-driving difficulties over time.

## Conclusions

This study provides evidence of the relationship between dry eye and night-driving vision by incorporating both objective and subjective measurements of dry eye symptoms. The findings suggest that drivers with poor tear film stability and severe dry eye symptoms may face considerable challenges when driving at night. Given the increasing demands of modern lifestyles that often require nighttime driving, addressing dry eye disease through targeted interventions could enhance the safety and well-being of affected individuals. Future research should further explore this relationship and assess the effectiveness of various therapeutic approaches to improve tear film stability and alleviate dry eye symptoms, ultimately enhancing night driving experiences for affected drivers.
